# Transcutaneous Auricular Vagus Nerve Stimulation for Treating Emotional Dysregulation and Inflammation in Common Neuropsychiatric Disorders

**DOI:** 10.3390/brainsci16010008

**Published:** 2025-12-20

**Authors:** William J. Tyler

**Affiliations:** 1 Center for Neuroengineering and Brain Computer Interfaces, Department of Biomedical Engineering, School of Engineering, University of Alabama at Birmingham, Birmingham, AL 35294, USA; wjpt@uab.edu; 2 Heersink School of Medicine, University of Alabama at Birmingham, Birmingham, AL 35294, USA

**Keywords:** mental health, depression, anxiety, PTSD, inflammation, neuromodulation, vagus nerve, cognition, emotion, autonomic nervous system

## Abstract

Development of new therapeutic approaches and strategies for common neuropsychiatric disorders, including Major Depressive Disorder, anxiety disorders, and Post-Traumatic Stress Disorder, represent a significant global health challenge. Recent research indicates that emotional dysregulation and persistent inflammation are closely linked and serve as key pathophysiological features of these conditions. Emotional dysregulation is mechanistically coupled to locus coeruleus and norepinephrine (LC-NE) or noradrenergic system activity. Stemming from chronic stress, persistently elevated activity of the LC-NE system leads to hypervigilance, anxious states, and depressed mood. Concurrently, these symptoms are marked by systemic inflammation as indicated by elevated pro-inflammatory cytokines, and central neuroinflammation indicated by microglial activation in brain regions and networks involved in mood regulation and emotional control. In turn, chronic inflammation increases sympathetic tone and LC-NE activity resulting in a vortex of psychoneuroimmunological dysfunction that worsens mental health. Transcutaneous auricular vagus nerve stimulation (taVNS) in a non-invasive neuromodulation method uniquely positioned to address both noradrenergic dysfunction and chronic inflammation in neuropsychiatric applications. Evidence spanning the past decade demonstrates taVNS works via two complementary mechanisms. An ascending pathway engages vagal afferents projecting to the LC-NE system in the brain stem, which has been shown to modulate cortical arousal, cognitive function, mood, and stress responses. Through descending circuits, taVNS also modulates the cholinergic anti-inflammatory pathway to suppress the production of pro-inflammatory cytokines like TNF-α and IL-6 mitigating poor health outcomes caused by inflammation. By enhancing both central brain function and peripheral immune responses, taVNS has shown significant potential for recalibrating perturbed affective-cognitive processing. The present article describes and discusses recent evidence suggesting that taVNS offers a promising network-based paradigm for restoring psychoneuroimmunological homeostasis in common neuropsychiatric conditions.

## 1. Introduction

Common neuropsychiatric disorders including Major Depressive Disorder (MDD), anxiety disorders, and Post-Traumatic Stress Disorder (PTSD) affect hundreds of millions of individuals worldwide, with prevalence rates of approximately 4–6% each. Collectively, these conditions are leading contributors to global disability and impose a substantial economic burden, with annual costs reaching hundreds of billions of dollars due to healthcare expenditures and lost productivity [1,2,3,4]. Although conventional treatments such as established pharmacotherapies and psychotherapies are available, efficacy is often limited to partial relief and high rates of treatment resistance. This leaves many individuals suffering from persistent, debilitating neuropsychiatric symptoms. This enduring clinical challenge underscores a critical need for alternative, network-based therapeutic strategies capable of addressing the core, interconnected pathophysiological mechanisms underlying these conditions. Emerging research has identified two critical, deeply entangled network dysfunctions that are exacerbated in these mental health conditions thereby presenting interesting therapeutic targets. Profound emotional dysregulation and chronic, low-grade neuroinflammation are common clinical features of depression, anxiety, and PTSD [5,6,7,8,9,10,11,12].

The locus coeruleus (LC) acts as the brain’s principal source of norepinephrine (NE) or noradrenaline (NA) and functions as a fundamental regulator of arousal, attention, learning, and the stress response [13,14,15,16,17]. The LC-NE system is highly responsive to threat, and under conditions of chronic stress, can become maladaptive leading to hyperactivity and hypersensitivity of LC neurons [14,16,17]. This hyperactive state is closely linked to the persistent anxious states, hyperarousal, and exaggerated startle responses seen across anxiety disorders and PTSD [14,16,17]. Given strong pharmacological evidence suggesting that modulating NE neurotransmission is a necessary component for effective antidepressant treatment [18], therapeutic focus is increasingly shifting toward complex system-level dysregulation of the LC-NE axis. This requires an examination of new therapeutic strategies beyond serotonin-centric, pharmacological approaches.

A vast body of evidence also now implicates chronic, low-grade inflammation as a common pathophysiological pathway across mental health disorders [7,10,12,19]. Patients with MDD, anxiety, and PTSD consistently exhibit elevated peripheral and central levels of key pro-inflammatory cytokines, including interleukin-1 beta (IL-1β), interleukin-6 (IL-6), and tumor necrosis factor-alpha (TNF-α) [12,19,20]. This systemic inflammation extends into the central nervous system (CNS) as neuroinflammation, characterized by the activation of resident immune cells, microglia [21,22,23]. This microglial activation can be visualized in vivo using Positron Emission Tomography (PET) imaging targeting the translocator protein 18 kDa (TSPO). Studies have demonstrated significantly elevated TSPO levels in mood control brain regions of depressed individuals [10,24]. This inflammatory milieu can disrupt synaptic plasticity, impair neurogenesis, and contribute to network dysfunction, suggesting that interventions capable of restoring neuroimmune balance offer a critical, novel therapeutic avenue [7,10,11,12,22]. This evolving psychoneuroimmunological understanding establishes a framework for the investigation of neuromodulation methods, such as transcutaneous auricular vagus nerve stimulation (taVNS), which offers a unique potential to address these dual pathologies simultaneously [25,26].

Vagus nerve stimulation (VNS) can involve surgically implanted electrodes used to treat epilepsy and depression. Several methods of noninavsive VNS (nVNS) have also been shown to produce effects on brain activity and immunological function. Of particular interest here is a taVNS which is a noninvasive method that targets the auricular branch of the vagus nerve (ABVN) and engages the brain through two primary, complementary mechanisms [25,27,28,29,30,31,32,33,34,35]. The first is an ascending pathway involves activation of vagal afferents projecting to the nucleus of the solitary tract (NTS), which directly modulates the activity of the LC-NE system, influencing central brain networks associated with arousal, attention, and cognitive control [25,27,28,29,30,31,32]. A second pathway involves the descending engagement of the cholinergic anti-inflammatory pathway (CAP), which is an autonomic reflex that inhibits peripheral inflammation by causing the efferent release of acetylcholine (ACh) [33,34,35,36]. ACh suppresses the production of pro-inflammatory cytokines like TNF-α and IL-6 via binding to alpha-7 nicotinic acetylcholine receptors (α7nAChR) on immune cells [19,35,36,37]. By simultaneously targeting the dysregulated LC-NE stress response and the pervasive inflammatory pathways, taVNS offers a unique paradigm for restoring both neuronal and immunological homeostasis in neuropsychiatric disorders [19,25,26,37]. This article offers a critical review of recent taVNS research while addressing unresolved questions and outlining future directions for its application in treating common neuropsychiatric disorders.

## 2. Psychophysiological Arousal and Noradrenergic Activity in Anxiety and Depression

Emotional dysregulation and neuroinflammation are two convergent pathophysiological pathways that contribute significantly to the symptomatology of depression, anxiety, and PTSD. These are not separate phenomena but rather interconnected processes that create a vicious cycle of dysfunction. The LC-NE system is a fundamental component of the brain’s arousal and stress-response networks. Nearly 250 years ago, scientists first identified the LC, a small, bilateral nucleus located in the dorsal pontine tegmental area of the brainstem [17]. Despite its small size, the LC is a powerhouse controlling neurophysiological arousal. It contains a cluster of NE neurons which have widespread projections throughout the entire CNS, from the spinal cord to the cerebral cortex [17].

**Figure 1:**
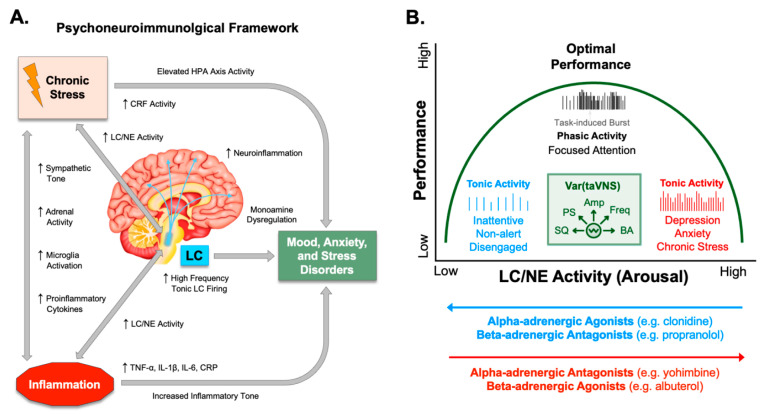
Interactions among chronic stress, inflammation, and locus coeruleus activity in emotional health and cognition. (**A**). The illustration depicts a psychoneuroimmunological framework where the interactions of chronic stress, inflammation, and locus coeruleus (LC) activity can lead to mental health dysfunction. Chronic stress increases LC and norepinephrine (NE) activity, as well as elevates hypothalamic–pituitary–adrenal (HPA) axis activity including increased release of C-reactive protein (CRP). Inflammation causes increased LC/NE activity and increases proinflammatory cytokines, such as tumor necrosis factor alpha (TNF-α), interleukin 1 beta (IL-1β), interleukin 6 (IL-6), and c-reactive protein, as well as activates microglia. (**B**). Cognitive performance can be described by an inverted-U function of locus coeruleus LC/NE activity. It has been shown that low-frequency tonic activity of LC neurons occurs during periods of inattentiveness, while higher frequency tonic activity of LC neurons occurs during periods of hyperarousal and poor attention leading to anxiousness and depressive symptoms. Pharmacological manipulation of alpha- and beta-adrenergic receptor (AR) activity influences LC/NE activity across this performance curve in different manners as shown. Other data have shown transcutaneous auricular vagus nerve stimulation (taVNS) can also tune arousal by modulating LC/NE activity as a function of several different stimulus variables (green inset). These taVNS variables that differentially influence autonomic arousal and LC/NE activity include: Stimulus Quality (SQ) including electrode coupling methods, human factors, stimulus comfort, and sensory intensity; Pulse Shape (PS) parameters such as biphasic, monophasic, interphase gap, pulse width, pulse symmetry and charge balance; Stimulus Amplitude and Current Density (Amp); Stimulus Frequency (Freq) ranging from low Hz to tens of kHz; and Baseline Arousal (BA), stress level, physical fatigue, and cognitive state.

As the main source of NE in the CNS, the LC is crucial for regulating arousal, attention, stress responses, sleep/wake cycles, learning, and memory, particularly for emotionally salient and threat-related events [13,14,16,17]. The LC-NE system is functionally modular with discrete regions and neuronal populations enabling both specific behavioral modulation and general arousal regulation while robust, aversive stimuli can trigger unified responses [13,14,16,38]. In response to chronic stress, this system can become dysregulated, leading to tonic hyperactivity and hypersensitivity of LC neurons (Figure 1A).

Studies have shown that corticotropin-releasing factor (CRF) containing afferents originating in the central nucleus of the amygdala provide direct synaptic inputs to dendrites of NA neurons in the LC [39,40,41]. This synaptic circuitry serves to consolidate the emotional and cognitive components of the stress response, tightly linking the perception of a threat with the physiological state of arousal [42]. Direct electrical, chemical, or optogenetic activation of the LC produces significant increases in anxiety and fear-like behavior in both rodents and monkeys [43,44,45]. In fact, panic symptoms can be induced with optogenetic or electrical LC activation, as well as with pharmacological agents that increase LC-NE activity [16,17,43,45]. These maladaptive states are closely linked to the hyperarousal, exaggerated startle responses, and persistent anxious states characteristic of anxiety disorders and PTSD. Importantly this network highlights a critical feedback loop where the amygdala’s threat processing and CRF-mediated chronic stress response can increase tonic LC activity, which in turn promotes anxiety (Figure 1A) [40,46,47].

The involvement of the LC-NE system in depression is not a simple matter of too much or too little NE. Instead, evidence highlights a complicated dysregulation of the entire system [18]. The function of LC-NE signaling often follows an inverted U-shape function, where both low and high rates of tonic LC neuron firing are associated with cognitive and emotional dysfunction (Figure 1B). Optimal performance occurs only when moderate, phasic firing rates [13,48]. Importantly, this indicates that both the timing and pattern of NE release, not just the quantity, are crucial for healthy brain function. Dysregulation of NA signaling can directly contribute to cognitive symptoms of depression, such as poor concentration and memory deficits [17,49,50].

Additional robust evidence supporting the role of LC-NE activity in depression comes from pharmacology. For decades, chronic treatment with virtually all classes of antidepressants including older monoamine oxidase inhibitors (MAOIs), adrenergic receptor (AR) agonists and antagonists, and tricyclic antidepressants to newer, more selective compounds has been shown to exert some effect on the NE system. For example, chronic treatment with antidepressants like imipramine and reboxetine primarily acts by increasing synaptic NE concentrations through reuptake inhibition implying NE neurotransmission is a necessary component for effective antidepressant treatment [18,51]. The actions of LC/NE signaling through both α-AR and β-AR subtypes has been shown to underlie symptoms in depression, anxiety, and PTSD (Figure 1B) [52,53,54,55,56,57,58]. Human studies have shown β-AR agonists and α_2_-AR antagonists can trigger frequent and intense panic attacks in individuals with panic disorder [59,60,61]. In contrast, clonidine, an α_2_-AR agonist, has been shown to alleviate anxiety symptoms and panic in certain contexts (Figure 1B) [62,63].

PET imaging studies have shown elevated platelet and brain expression levels of the α_2_-AR in depressed patients and depressed suicide victims compared to healthy controls [64,65,66]. Treatment with tricyclic antidepressants decreases the elevated α_2_-AR expression levels observed by PET imaging in depression [64,67]. The α_2A_-AR has been shown to play a protective role in rodent models of depression and anxiety [68]. Other evidence from animal models suggests the α_2C_-AR may also be a promising therapeutic target for depression [69]. Patients with depression especially those exhibiting psychomotor agitation have shown decreased β-AR function as indicated by significantly lower isoproterenol-stimulated cyclic AMP levels compared to controls [70,71,72]. In anxiety disorders, patients diagnosed with panic disorder or agoraphobia with panic attacks demonstrate lower number of lymphocyte β-AR binding sites coupled with a higher affinity of binding compared to healthy subjects [73]. Furthermore, β-AR is demonstrably involved in stress-related behavioral changes, as studies show that the β-AR antagonist propranolol prevents anxiety-like behavior induced by repeated social defeat in mice and attenuates stress-induced changes in defensive withdrawal in rats [55,74]. Together, these findings underscore that LC-NE dysfunction in neuropsychiatric dysfunction reflects a complex and critical imbalance in NA signaling dynamics.

## 3. Inflammatory Signatures of Anxiety, Stress, and Mood Disorders

Across major psychiatric disorders, a distinct inflammatory signature has emerged, characterized by elevated biomarkers and specific patterns of brain changes. A growing body of evidence now implicates chronic, low-grade inflammation as a common pathophysiological pathway in mental health disorders. The influence of inflammation on mental health originates in the periphery, where the immune system first detects a threat. The immune system is activated by different molecular signals that indicate either an external threat (not self) or internal damage (self). Pathogen-Associated Molecular Patterns (PAMPs) are conserved molecules derived from microorganisms that signal a pathogen intrusion. A classic example is lipopolysaccharide (LPS), a component of the outer membrane of Gram-negative bacteria, which is a potent inducer of inflammation [75,76,77,78]. Damage-Associated Molecular Patterns (DAMPs) are endogenous molecules released from cells during tissue injury, trauma, or metabolic stress. A key example is adenosine triphosphate released from damaged cells [75,76,77,78]. Whether triggered by PAMPs or DAMPs, these molecular patterns activate toll-like receptors (TLRs) and critical transcription factors, such as nuclear factor kappa B (NF-κB). Once activated, NF-κB promotes the transcription of genes responsible for producing proinflammatory cytokines, including TNF-α, IL-1β, and IL-6 [75,76,77,78]. The initial release of these cytokines triggers the production of even more, creating a self-amplifying feedback loop known as the cytokine cascade or cytokine storm.

**Figure 2:**
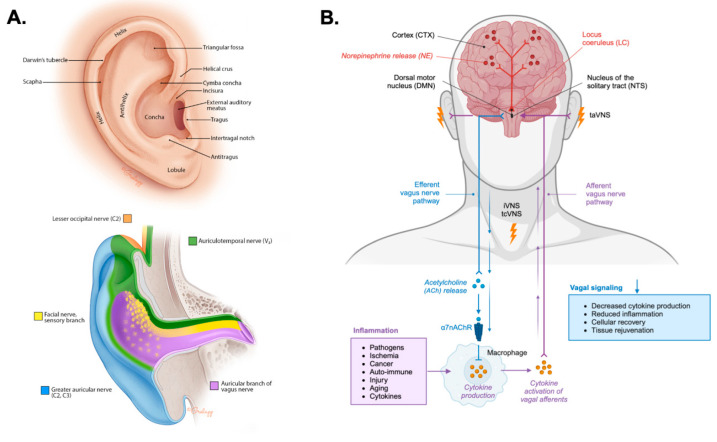
Neuroanatomy of transcutaneous auricular vagus nerve stimulation. (**A**). The anatomical illustration depicts the anatomy of the human external ear showing prominent structures (top). The illustration (bottom) depicts the sensory innervation of the external ear by the lesser occipital nerve (C2; orange), the great auricular nerve (C2, C3; blue), the facial nerve (yellow), the auriculotemporal nerve (ATN) or third branch of the trigeminal nerve (V3; green), and the auricular branch of the vagus nerve (ABVN; purple). The images in panel A were reproduced from reference [95] with permission from the illustrator Chris Gralapp. (**B**). A schematic circuit representation of transcutaneous auricular vagus nerve stimulation (taVNS), transcutaneous cervical vagus nerve stimulation (tcVNS), and surgically invasive vagus nerve stimulation (iVNS) targeting vagal afferents projecting to the nucleus tractus solitarius (NTS), locus coeruleus (LC), and dorsal motor nucleus (DMN). Norepinephrine (NE) release from the LC is shown projecting to large scale brain networks. Efferent vagal signaling engages the cholinergic anti-inflammatory pathway, where acetylcholine (ACh) release activates α7 nicotinic acetylcholine receptors (α7nAChR) on immune cells (macrophages, dendritic cells, T and B lymphocytes, and mast cells) reducing pro-inflammatory cytokine production to promoting cellular recovery and tissue restoration.

Inflammation is signaled to the brain through several mechanisms including a humoral and neural pathway. The humoral pathway involves the circulation of cytokines and other inflammatory mediators in the bloodstream. While large molecules like TNF-α and IL-1β do not readily diffuse across the blood–brain barrier (BBB). They can leak across damage or compromised BBB, they can be actively transported across the BBB, they can affect circumventricular organs, and they can signal through endothelial messengers like prostaglandins and nitric oxide [79]. The neural pathway involves direct transmission of inflammatory signals to the brain via nerve afferents, with the vagus nerve serving as the primary conduit. The vagus nerve conveys afferent signals from a wide range of visceral, somatic, and immunological signals to the brain. These signals in turn activate microglia and astrocytes triggering neuroinflammation providing a direct link between systemic inflammation and the symptoms of mood disorders. The brain can actively regulate peripheral inflammation when it detects inflammation via vagal afferents. Through this cholinergic anti-inflammatory pathway (CAP) discussed further below, the brain sends signals back down the vagus nerve to suppress cytokine production in organs like the spleen, demonstrating a bidirectional neuroimmune reflex loop (Figure 2B) [34,36].

Patients with depression, anxiety, and PTSD consistently show elevated peripheral and central levels of pro-inflammatory cytokines, including IL-1β, IL-6, TNF-α in both blood and cerebrospinal fluid (Figure 1A) [6,7,8,12,19,80,81,82]. Elevated C-reactive protein (CRP) is frequently elevated in individuals with depression and anxiety as a function of symptom severity [6,83,84]. At the cellular level in the brain, neuroinflammation involves the activation of microglia. TSPO has been established as a key biomarker of microglial activation that can be measured in the living human brain using PET imaging. PET studies have demonstrated significantly elevated TSPO levels in critical mood-regulating brain regions, such as the prefrontal cortex and anterior cingulate cortex, of individuals experiencing a major depressive episode [10,24,85]. Inflammation influences other critical physiological systems that affect mood and behavior. For example, proinflammatory cytokines can directly stimulate the hypothalamic–pituitary–adrenal (HPA) axis, contributing to the hypercortisolemia commonly observed in depression [86,87,88]. Other recent evidence underscores the fundamental role of the gut–brain axis, mediated by the vagus nerve, in neuropsychiatric health. Perturbations in the diversity and functionality of the gut microbiota have been shown to trigger inflammatory responses that influence mood and behavior [89,90]. Reflecting these actions, diets high in sugar including soft drink consumption have been shown to elicit depression and anxiety symptoms by increasing inflammation driven by gut microbiota alterations [91,92,93,94].

Together, the dysregulated LC-NE system and inflammatory pathways described above represent prime targets for therapeutic intervention. Addressing these dual pathologies has been a major challenge for conventional pharmacotherapy, which often targets one system at the expense of the other. As discussed below, this presents a major opportunity for using taVNS to engage both dysfunctional pathways simultaneously in the treatment of depression, anxiety, and PTSD (Figure 2B and Table 1).

## 4. Anatomical Basis for taVNS

**Figure 3:**
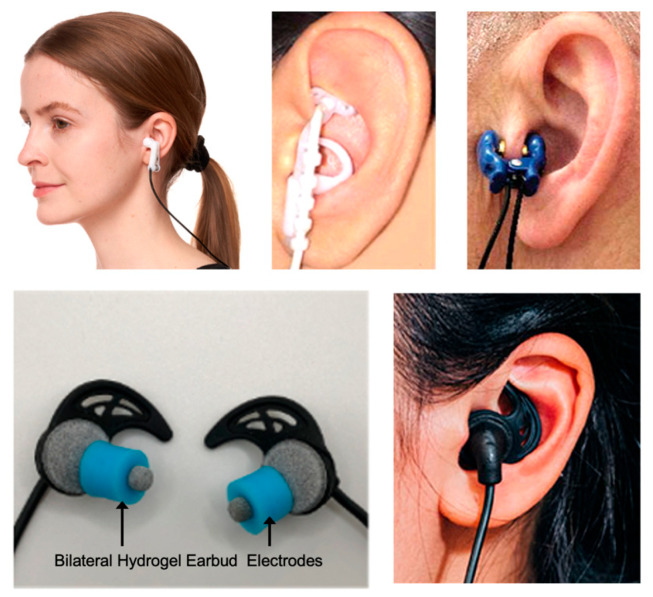
Methods of transcutaneous auricular vagus nerve stimulation. Several common methods of conducting transcutaneous auricular vagus nerve stimulation (taVNS) or transcutaneous electrical nerve stimulation of the external ear are shown. Electrode ear clips (top left and top right) can be affixed the tragus while customized electrode assemblies can deliver stimuli to the concha (top middle). Bilateral hydrogel earbud electrodes (BRAIN Buds, IST, LLC) can also be used to target afferent vagal and trigeminal fibers lining the external acoustic meatus as shown in the bottom panel of images. Methods of taVNS often include stimulation across a range of intensities typically less than 4 mA at frequencies ranging from 10 to 20,000 Hz for durations lasting 10 to 30 min per session. Some applications may use reinforcement-based stimulation in which only a brief 0.5 to 3 s stimulus is given during task training or rehabilitation [25,28,29,31,107].

Understanding the specific anatomy of the external ear is important for the application and efficacy of taVNS. The success of taVNS is entirely dependent on the precise targeting of the cutaneous distribution of the ABVN. The location and density of these nerve fibers determine where stimulation should be applied to ensure that the electrical signal is transmitted to the CNS to produce its therapeutic effects. The sensory nerve supply of the human auricle is characterized by a heterogeneous distribution of cranial and cervical nerves (Figure 2A). Cadaveric studies have meticulously mapped these distributions, revealing that the ABVN predominantly innervates specific regions of the external ear. The cymba conchae is innervated by the ABVN in all cases [27,96]. The innervation of the cavity of the conchae is more variable where in 45% of specimens, the ABVN solely innervates this region and in the remaining 55%, it receives dual innervation from both the ABVN and the great auricular nerve (GAN) [96]. Other areas, such as the tragus and antihelix, show significant overlap with other nerves, including the GAN and the auriculotemporal nerve (ATN) branch of the trigeminal nerve [96]. The colocation of vagal and trigeminal nerves highlights their shared ascending pathways to the brainstem through the spinal trigeminal sensory nuclear complex, as well as common descending pathways along the brain-heart axis as observed through the trigeminocardiac reflex and mammalian diving reflex [97,98,99,100,101,102,103]. The external acoustic meatus (EAM) is also innervated by the ABVN and ATN, as well as the facial nerve [96,104,105,106]. Methods and device for transcutaneous electrical nerve stimulation (TENS) of the external ear are generally referred to collectively as taVNS although different degrees of cranial and cervical nerve afferents may be influenced. Beyond nerve targeting considerations, the structural features of the external ear require different human factors and biomedical engineering approaches to conduct taVNS safely, reliably, and comfortably for users [25] (Figure 2A and Figure 3).

The therapeutic effects of taVNS are mediated through a well-characterized central afferent pathway. Electrical stimulation of the ABVN on the external ear transmits signals along vagal afferents to the NTS in the brainstem (Figure 2B). The NTS serves as a key central vagal relay center, integrating sensory information and projecting to several higher-order brain structures. Among its most critical projections are those to the LC. The NTS and LC are considered key targets for the therapeutic effects of taVNS [27]. This direct anatomical link between the periphery and key brainstem nuclei provides a direct conduit to the LC and interconnected brainstem nuclei [29,30,31], whose dysregulation in response to chronic stress and inflammation contributes to the pathophysiology of these disorders [13,16,17,18,38].

## 5. Ascending Modulation of Noradrenergic Signaling by taVNS

The primary CNS mechanism of taVNS involves the ascending modulation of the LC-NE system (Table 1). As mentioned above the process begins with the transcutaneous electrical modulation of ABVN afferents, which transmit signals to the NTS in the brainstem. The NTS, in turn, projects directly to and modulates the firing rate and pattern of neurons within the LC. Several functional neuroimaging studies have shown that taVNS directly modulates blood oxygen-level dependent fMRI signals in the NTS and LC [30,108,109,110,111]. Norepinephrine receptor engagement by VNS has been demonstrated in vivo using PET imaging of ^11^C-yohimbine, a radiolabeled α_2_-AR antagonist. These investigations showed that acute VNS leads to a marked reduction in receptor binding, a finding consistent with the widespread release of NE in cortical, limbic, and thalamic brain regions [112]. Interestingly, it has been shown the local activation of α_2_-ARs is required for VNS-induced motor cortical plasticity [113] while β-AR blockade enhances it [114]. Human psychophysiological investigations have also demonstrated that taVNS activates NA signaling as observed through modulation of pupil diameter a known biomarker of LC-NE activity and cortical arousal [29,31,32]. Several other electroencephalography (EEG) and functional neuroimaging studies have shown large scale modulation of cortical networks in response to taVNS [107,108,109,115,116,117,118,119,120,121,122]. This modulation of the LC-NE system by taVNS has significant functional consequences, including enhanced arousal, heightened attention, and improved cognitive control—functions that are often impaired in neuropsychiatric disorders [25,115,118,123,124,125,126,127,128].

## 6. Modulation of the Descending Cholinergic Anti-Inflammatory Pathway by taVNS

The second major mechanism of taVNS is its ability to engage the CAP, a critical neuroimmune reflex circuit that regulates systemic inflammation (Table 1). Stimulation of afferent vagus nerve fibers triggers a descending, efferent vagal response that can inhibit peripheral inflammation (Figure 2B) [19,34,35,36,37]. This pathway is mediated by the release of ACh targeting the α7nAChR located on the surface of immune cells like macrophages, dendritic cells, T and B lymphocytes, and mast cells. In turn, activation of α7nAChR suppresses intracellular inflammatory signaling cascades by inhibiting the activation of NF-κB, as well as decreasing production and release of pro-inflammatory cytokines like TNF-α [19,34,35,36,37]. As discussed below several lines of evidence corroborate these mechanisms and demonstrate that taVNS can suppress inflammation by modulating NF-κB and cytokine signaling.

Additional studies across various animal models and human diseases of inflammation demonstrate that taVNS effectively modulates key inflammatory biomarkers, including CRP and numerous cytokines. The data show taVNS produces a consistent pattern of reducing pro-inflammatory and increasing anti-inflammatory markers. In human trials, taVNS significantly reduced post-surgical inflammation following lung lobectomy, decreasing serum concentrations of CRP and IL-6 while elevating IL-10 on the first postoperative day [129]. For patients with COVID-19, taVNS led to a significant reduction in both CRP and IL-6 [130]. In sepsis patients, taVNS resulted in significant reductions in serum pro-inflammatory cytokines like TNF-α and IL-1β, accompanied by increases in anti-inflammatory IL-4 and IL-10 [131]. For neurological conditions, taVNS has been shown to mitigate the inflammatory response after subarachnoid hemorrhage SAH, significantly reducing TNF-α and IL-6 in both plasma and cerebrospinal fluid [132]. In large vessel occlusion stroke patients, taVNS significantly reduced normalized aggregate pro-inflammatory cytokines and IL-6 levels [133]. In mouse models of colitis, taVNS decreased colonic pro-inflammatory markers such as IL-1β and TNF-α, while upregulating anti-inflammatory TGF-β and IL-10 [134]. In rat models of depression and anxiety, taVNS has been shown to decrease central and peripheral pro-inflammatory cytokines, including IL-1β and TNF-α, by inhibiting the activation of inflammatory pathways like NF-κB in the hypothalamus, hippocampus, and prefrontal cortex [135,136,137]. Presently, there is a lack of human studies that investigate whether taVNS can similarly reduce inflammation in depression and anxiety. Encouraging preliminary results have shown however that transcutaneous cervical nerve stimulation (tcVNS) can reduce IL-6 and interferon-γ in subjects with PTSD compared to healthy controls [138]. Future clinical trials examining the influence of taVNS on mental health should include the quantification of inflammatory biomarkers to expand knowledge in the field while addressing putative therapeutic targets.

The two distinct but complementary mechanisms, central neuromodulation and peripheral anti-inflammatory signaling, described above position taVNS as an exceptional method for addressing the core pathologies of emotional dysregulation and inflammation in neuropsychiatry. The sections below discuss evidence from studies translating these mechanistic actions into measurable changes in autonomic tone, affective processing, and clinical symptom severity.

## 7. Effects of taVNS on Autonomic Balance and Heart Rate Variability

An interesting therapeutic indicator of taVNS is its ability to rebalance autonomic homeostasis by suppressing sympathetic dominance of vagal cardiac tone. This modulation is frequently measured by improvements in heart rate variability (HRV), which is associated with better cardiac autonomic control and stress recovery. Studies have shown taVNS can produce significant increases in HRV metrics such as the root mean square of successive differences (RMSSD), the percentage of adjacent intervals differing by more than 50 msec (pRR50), and the high-frequency (HF) component, which collectively indicate a shift towards parasympathetic predominance under resting conditions [139,140]. It has also been shown to directly dampen sympathetic nervous system activation as measured during sympathetic muscle nerve activity recordings [140]. This vagal-mediated increase in HRV extends to experimental challenges and stressful conditions. For example, taVNS has been shown to increase HRV correlating with significant suppression of experimentally induced pain [141].

Other studies have shown taVNS can mitigate physiological responses to acute mental and psychosocial stress [142,143], and improves autonomic functioning in extreme environmental conditions, particularly when the stimulation is applied repeatedly [144]. The downstream consequences of this autonomic regulation are significant for the HPA axis and cognitive function. Notably, taVNS has been shown to inhibit the release of salivary cortisol during mental stress, suggesting therapeutic potential for chronic inflammatory conditions associated with glucocorticoid dysfunction [142]. Cognitively, taVNS can improve cognitive flexibility by reducing perseverative thinking following a psychosocial stressor [143], while repeated stimulation can enhance overall cognitive performance [144]. The efficacy of taVNS appears more dependent on the individual’s baseline physiological state than on the specific stimulation parameters used, provided the correct anatomical target is stimulated. This is supported by evidence that taVNS does not produce parameter-specific effects on HRV [139,145]. In contrast, individual variability is a powerful predictor of outcomes. For instance, a higher baseline sympathovagal balance (low-frequency to high-frequency ratio) predicts a greater decrease in this ratio [139], the degree of parasympathetic activation during stimulation predicts the subsequent magnitude of pain suppression [141], and taVNS is particularly effective at reducing perseverative thinking in individuals who exhibit greater autonomic inflexibility during a stressor [143]. The findings with respect to HRV are clinically relevant given low vagal tone is strongly associated with impaired recovery from stress, poor mental health, and is associated with a pro-inflammatory state further linking autonomic dysregulation to the symptoms observed in stress, mood, and anxiety disorders [26,146].

## 8. Modulation of Stress, Cognition, and Emotion by taVNS

Current evidence indicates that taVNS beneficially impacts cognition, with notable effects on executive functions [119,147,148,149]. More specifically, taVNS can enhance cognitive flexibility, particularly in conditions of high cognitive demand [144,147,150] and improve emotional inhibitory control by enabling more efficient suppression of prepotent emotional responses [151,152]. In addition to executive control, taVNS has demonstrated a causal link with the formation of emotional memories by increasing recollection-based memory specifically for emotionally relevant information [153]. Another study has shown taVNS can significantly improve visual memory [154]. This effect may extend to social memory and threat processing functions of the vagus [155,156]. Consistent with these functions, treatment with taVNS has been shown to mitigate negative emotional bias by improving the detection of positive facial expressions and reducing negative emotions [157]. Psychological markers of resilience are improved by taVNS as indicated by significant reductions in cognitive rigidity and perseverative thinking following a psychosocial stressor [143]. Similarly, taVNS also prevents increases in perceived stress following cognitively or physically demanding tasks [146,158]. These behavioral and cognitive outcomes highlight a targeted effect of taVNS on the brain’s arousal, emotional regulation, and attention systems.

The cognitive and emotional benefits of taVNS are also rooted in specific, measurable changes in brain activity and network dynamics. At a fundamental level, taVNS enhances cortical arousal and alertness by attenuating high-frequency alpha oscillations and reducing the duration of EEG microstate C, a neural signature associated with alertness [115], while also increasing power in the delta frequency band [159]. This general enhancement in cortical arousal provides the foundation for more targeted, network-level changes that support executive function. At the network level, taVNS promotes more efficient brain communication following a stress task as it enhances global network efficiency evidenced by a reduction in prefrontal alpha and theta band activity [158]. It also strengthens functional connectivity to support emotional control. This is achieved by reducing alpha band power spectral density and increasing phase locking within prefrontal networks, as well as enhancing neurofunctional coupling between the inferior and orbital frontal cortex [151,158]. This increased network efficiency translates into more parsimonious use of neural resources for cognitive control, as demonstrated by a significant reduction in the frontal N2 potential in NoGo tasks, which suggests fewer cognitive resources are needed to successfully inhibit a response [148,151]. Functional neuroimaging corroborates these findings, showing that taVNS modulates neural activity in key emotion regulation regions, including the bilateral precuneus and temporal gyrus, which is thought to facilitate the integration of emotional responses and memories [160].

The capacity of taVNS to reliably modulate cognitive, emotional, and neurophysiological systems gives it significant therapeutic potential for psychiatric disorders. More specifically, the ability of taVNS to modulate perseverative cognition [143], negative emotional bias [157], and inhibitory control [151] positions it as a promising therapeutic tool. Furthermore, the causal role of taVNS in modulating emotional memory formation suggests potential applications for disorders with altered memory functions, such as PTSD [153]. More broadly, its ability to improve emotional regulation by modulating prefrontal and temporal brain regions points to its utility in treating a range of conditions characterized by emotional dysregulation [107,109,116,122,151,160,161,162]. However, to translate this potential into clinical practice, future research must address several critical areas. There is a clear need for longitudinal studies with larger, more diverse samples to confirm the observed health benefits [28,119,144]. Concurrently, researchers must work to identify optimal stimulation parameters to maximize efficacy [25,29,119] and validate robust biomarkers that can illuminate its mechanisms of action and help predict therapeutic outcomes in individuals with neuropsychiatric diseases [25,144,163,164,165].

## 9. Clinical and Translational Efficacy of taVNS in Depression, Anxiety, and PTSD

Clinical trial data indicate taVNS is an effective approach for reducing symptoms of MDD. Studies have shown taVNS treatment produces significant decreases in depression scores in depressed subjects as measured with the Hamilton Depression Rating Scale (HAM-D), Beck Depression Inventory (BDI), and other clinically valid scales [126,128,166,167,168,169,170,171]. When directly compared to pharmacotherapy, taVNS demonstrated therapeutic effects comparable to citalopram, which is a selective serotonin reuptake inhibitor. However, patients treated with taVNS experienced significantly higher remission rates at weeks four and six than those treated with citalopram [167]. Li et al. (2022) also showed that taVNS treatment decreased plasma serotonin, NA, and γ-aminobutyric acid (GABA) while increasing dopamine producing effects like citalopram on these neurotransmitters [167]. Enhancing fear extinction is a critical goal in PTSD treatment. It has been shown that taVNS paired with extinction training strengthens the consolidation of fear extinction memory [121,172]. A recent pilot study demonstrated that taVNS treatment of subjects diagnosed with PTSD can significantly improve sleep quality (depth) and autonomic function which are typically disrupted by the disorder [173]. Other evidence from the study of VNS in the treatment of PTSD suggests it provides a viable treatment option worthy of further investigation [138,174,175,176,177]. Translational investigations of the use of taVNS for the treatment of anxiety have also shown encouraging results. Several recent feasibility and exploratory clinical trials have reported that taVNS treatment can significantly reduce anxiety across different health and experimental conditions as measured by the Generalized Anxiety Disorder-7 (GAD-7), Beck Anxiety Inventory (BAI), and other scales [170,178,179,180,181]. For example, a feasibility study demonstrated that at-home taVNS improved anxiety scores in youth with comorbid autism spectrum disorder and anxiety [182]. While these initial results on depression, PTSD, and anxiety are encouraging, there remains a need to conduct larger randomized controlled trials with a focus on gaining regulatory approvals for neuropsychiatric indications.

Beyond symptom reduction, taVNS can also induce specific behavioral and cognitive improvements. In patients with MDD, it has been shown to enhance motivation, invigorate effort, and increase the subjective wanting of rewards [118,146,183], as well as improve emotional processing by increasing accuracy in detecting positive facial expressions [157]. For individuals with high trait anxiety, taVNS has been found to reduce negative thought intrusions and perseverative thinking [143,184]. These targeted benefits are complemented by broader improvements in quality of life, including better sleep [185,186,187,188] and a reduction in pain perception, a common comorbidity in psychiatric conditions [141,179]. Neuroimaging findings further illustrate that taVNS modulates activity across large-scale brain networks implicated in psychiatric disorders [120,161,189,190,191]. These effects include the normalization of functional connectivity within the limbic system, particularly the amygdala, in patients with depression [191], and the regulation of the cortico-striatal-thalamic-cortical circuit, evidenced by the downregulation of hyperactivity in the ventral striatum [192]. Furthermore, taVNS has been shown to alter connectivity between core cognitive control and emotional processing networks, including the default mode network (DMN), frontoparietal network (FPN), and cingulo-opercular network (CON) [107,109,116,122,161,162]. These findings collectively support the therapeutic potential of taVNS, demonstrating its ability to appropriately modulate neurophysiological outcomes and improve clinical symptoms across a spectrum of common neuropsychiatric disorders.

## 10. Conclusions

The taVNS method is a promising, non-invasive therapeutic modality that directly addresses the core, interconnected pathologies of emotional dysregulation and neuroinflammation in neuropsychiatric disorders. Through its dual mechanisms of action, taVNS modulates the central locus coeruleus-norepinephrine system to enhance cognitive control and emotional processing, while simultaneously engaging the descending cholinergic anti-inflammatory pathway to suppress peripheral inflammation. This unique combination of effects allows taVNS to restore autonomic and immune homeostasis, leading to measurable improvements in clinical symptoms. In contrast to pharmacological agents, which may indiscriminately modify LC-NE activity and cause unintended side effects, taVNS promotes physiological homeostasis by balancing arousal, stress, and inflammation throughout various functional systems. The convergence of evidence suggests that taVNS may not merely treat symptoms, but rather fundamentally restores the organism’s capacity for adaptive allostasis, a crucial insight for developing preventative and resilience-building neuromodulatory strategies. While the existing evidence is compelling, future research is essential to fully realize its clinical potential.

The clinical potential of taVNS derives from its capacity to simultaneously address two interacting biological drivers of mood and anxiety disorders. A unified model of action proposes that by modulating the hyperactive LC-NE alarm system via its ascending pathway, taVNS normalizes central arousal and stress reactivity. At the same time, its descending pathway activates the cholinergic anti-inflammatory reflex to reduce systemic pro-inflammatory signals implicated in the pathophysiology of depression and anxiety. These two mechanisms likely interact to produce specific therapeutic effects. For instance, the simultaneous downregulation of the hyper-aroused LC-NE system likely contributes directly to the observed attenuation of sympathetic dominance and blunting of HPA axis stress reactivity described above. Concurrently, the reduction in systemic pro-inflammatory cytokines, which are known to impair neuroplasticity, may provide the neurochemical environment necessary for enhancing fear extinction memory and recalibrating negative affective biases. By calming a hyper-responsive central stress system while also dampening peripheral inflammation, taVNS may disrupt the vicious cycle where stress promotes inflammation and inflammation, in turn, exacerbates stress and mood dysregulation.

Despite the promising findings, the field of taVNS research faces several methodological challenges that must be addressed to establish its clinical utility robustly. Many of the reviewed studies were pilot or exploratory trials with small sample sizes, which limits the generalizability of their findings and increases the risk of false positives. Furthermore, designing an inert but credible sham control is a significant challenge in neuromodulation research. While some studies use stimulation of a non-vagal site like the earlobe, the complete blinding of participants can be difficult to achieve, as active stimulation often produces a noticeable sensation. This complicates the interpretation of results and the separation of true therapeutic effects from placebo responses. Finally, there is wide variability in the stimulation parameters (e.g., frequency, pulse width, intensity, duration) used across studies, which makes it difficult to compare results between trials and to determine the optimal settings for specific clinical indications. While methodological challenges remain, continued research focused on parameter optimization, biomarker identification, and large-scale clinical trials holds the promise of establishing taVNS as a scalable and accessible tool in the clinical management of common neuropsychiatric disorders.

## Figures and Tables

**Table 1:** Dual Mechanisms of Action of Transcutaneous Auricular Vagus Nerve Stimulation.

Mechanistic Pathway	Neurobiological and Physiological Effects
Ascending Neuromodulation	Transcutaneous auricular vagus nerve stimulation (taVNS) activates afferent vagal fibers from the external ear projecting to the nucleus of the solitary tract (NTS). The NTS then modulates activity in the locus coeruleus (LC), leading to widespread release of norepinephrine (NE). This helps to stabilize mood and arousal, as well as enhance attention, cognitive control, and facilitates neuroplasticity related to learning and memory.
Descending Anti-Inflammatory Modulation	Afferent vagal activation triggers the efferent cholinergic anti-inflammatory pathway (CAP). Activation of the CAP involves the release of acetylcholine (ACh), which binds to α7 nicotinic ACh receptors (α7nAChR) on peripheral immune cells like macrophages to inhibit the activation of NF-κB and production of pro-inflammatory cytokines such as TNF-α and IL-6.

## Data Availability

No new data were created or analyzed in this study.

## Abbreviations

The following abbreviations are used in this manuscript:

ABVN	Auricular Branch of the Vagus Nerve
ACh	Acetylcholine
Amp	Current Amplitude
AR	Adrenergic Receptor
ATN	Auriculotemporal Nerve
BA	Baseline Arousal
BAI	Beck Anxiety Inventory
BBB	Blood–Brain Barrier
BDI	Beck Depression Inventory
CAP	Cholinergic Anti-inflammatory Pathway
CNS	Central Nervous System
CON	Cingulo-Opercular Network
CRF	Corticotropin-Releasing Factor
CRP	C-Reactive Protein
DAMP	Damage-Associated Molecular Pattern
DMN	Dorsal Motor Nucleus or Default Mode Network (context dependent)
EAM	External Acoustic Meatus
EEG	Electroencephalography
FPN	Frontoparietal Network
Freq	Frequency
GABA	Gamma-Aminobutyric Acid
GAD-7	Generalized Anxiety Disorder-7
GAN	Great Auricular Nerve
HAM-D	Hamilton Depression Rating Scale
HF	High Frequency (HRV component)
HPA	Hypothalamic–Pituitary–Adrenal (axis)
HRV	Heart Rate Variability
IL-1β	Interleukin-1 beta
IL-4	Interleukin-4
IL-6	Interleukin-6
IL-10	Interleukin-10
iVNS	Invasive Vagus Nerve Stimulation
LC	Locus Coeruleus
MAOIs	Monoamine Oxidase Inhibitors
MDD	Major Depressive Disorder
NA	Noradrenaline
NE	Norepinephrine
NF-κB	Nuclear Factor kappa-B
NTS	Nucleus Tractus Solitarius/Nucleus of the Solitary Tract
PAMP	Pathogen-Associated Molecular Pattern
PET	Positron Emission Tomography
pRR50	Percentage of NN Intervals > 50 msec
PS	Pulse Shape
PTSD	Post-Traumatic Stress Disorder
RMSSD	Root Mean Square of Successive Differences
SAH	Subarachnoid Hemorrhage
SQ	Stimulus Quality
taVNS	Transcutaneous Auricular Vagus Nerve Stimulation
tcVNS	Transcutaneous Cervical Vagus Nerve Stimulation
TENS	Transcutaneous Electrical Nerve Stimulation
TGF-β	Transforming Growth Factor-beta
TLR	Toll-Like Receptor
TNF-α	Tumor Necrosis Factor-alpha
TSPO	Translocator Protein (18 kDa)
VNS	Vagus Nerve Stimulation
α7nAChR	Alpha-7 Nicotinic Acetylcholine Receptor
α-AR	Alpha-Adrenergic Receptor
β-AR	Beta-Adrenergic Receptor
